# Switching gears for the sustainable development goals

**DOI:** 10.1038/s41467-021-24286-3

**Published:** 2021-07-05

## Abstract

In the face of an on-going global pandemic and the growing urgency of climate change, the challenge of building an equitable and sustainable world has never been greater. Thus, now more than ever, we want to support and highlight research efforts made at attaining the UN sustainable development goals.

In 2015 the sustainable development goals (SDGs) were set by the UN to tackle endemic issues in how humanity can live both sustainably and fairly. A total of 17 goals were agreed upon ranging from eliminating poverty, inequality and hunger to taking action on climate change. After almost six years and the upheavals to everyday life since, the need to attain these goals by 2030 remains ever more urgent. Despite the brief respite in global emissions of CO_2_ during national lockdowns in 2020, these have bounced back to even higher levels^1^. Covid-19 has exacerbated existing social and health inequalities in many countries^2^, and vaccine allocation and purchase has resulted in shortages for developing nations. And although there is a continuing decline in extreme poverty, this is slowing down^3^. Globally, one in three people do not have access to clean, safe drinking water, and around 3 billion people still lack basic hand washing facilities^4^. Scientific research must switch gears and place addressing inequality issues at the forefront of its activities^5^. While it is unclear if all SDGs will be reached by 2030, they remain a vital framework towards making global progress towards equality.

While it is unclear if all SDGs will be reached by 2030, they remain a vital framework towards making global progress towards equality.

To be met, the SDGs need research that not only highlights the current problems, but also proposes solutions. While applied sciences and technology will be part of the solution, technology alone will not be enough. After all, some solutions proposed seem simple enough in theory: switch to electric vehicles, reduce CO_2_ emissions, reduce fishing, stop all deforestation. This view neglects, however, that many current practices are necessary for a large part of the global population to fulfill their basic needs. Thus, solutions have to be adapted and developed in a manner that is sustainable for all populations and all countries - these are likely to be complex and costly. While some of the SDGs require technological solutions, most will need systematic transformations of societies to address income and gender inequality or building sustainable institutions. A truly sustainable world is one that is equitable and just.

The problems the world faces are complex, multifaceted and unlikely to be solved by a single solution, a solitary initiative or one point of view. More than ever, scientific fields need to talk not to each other but with each other, along with extending the conversation to policy makers, stakeholders and the communities impacted by the challenges the world faces. One of the keys to achieving the SDGs will be interdisciplinary, multidisciplinary and transdisciplinary research bringing together different ideas and approaches from across the sciences. While the necessity of such approaches is increasingly acknowledged, many studies are still predominantly contained within the borders of traditional disciplines. Nor can any single one of the 17 SDGs be separated from the others, as the goals of each are bound to the progress of them all. For instance, achieving Zero hunger (SDG 2) requires significant progress in reduction in poverty (SDG 1), action on climate change to reduce weather extremes (SDG 13) and protection of life on land and water (SDG 14 and 15). As a multidisciplinary journal, we want to support further the relevant research.

In a first step, we are launching a series of thematic collections that brings together content from across the journal in the physical, Earth, life, health and social sciences. Each collection will highlight research and opinion to explore the causes, impacts and solutions for translation into policy. By bringing this research from across the breadth of the sciences together, we want to highlight that living in a sustainable and fair world is not just the preserve of any single field of research, but an opportunity for diverse communities of researchers to embrace addressing the challenges by working together.

We are pleased to launch this series in the coming weeks with *Clean Air*. This curated collection of research, commentaries and interviews will explore the complex problem of air pollution and how using the SDGs framework could be part of the solution. In upcoming SDG thematic collections, as we explore the challenges facing the world - ranging from the food we eat, the energy we need, and to the protection of the land and water around us - and ultimately how to achieve a sustainable and equitable economy and society, we invite our authors, reviewers and readers to consider their work in the context of the SDGs and how it can contribute to accomplishing these goals.
